# Role of Anticoagulants for Stroke Prevention in Low-Risk Population Having Atrial Fibrillation and Chronic Kidney Disease: A Systematic Review

**DOI:** 10.7759/cureus.31364

**Published:** 2022-11-11

**Authors:** Niriksha Ravi, Rajita Ramaraju, Aastha Vats, Athira R Nair, Atithi K Bandhu, Divya Koirala, Manoj R Pallapothu, Maria G Quintana Mariñez, Mohana Chakkera, Ana P Arcia Franchini

**Affiliations:** 1 Internal Medicine and Neurology, California Institute of Behavioral Neurosciences & Psychology, Fairfield, USA; 2 Internal Medicine, California Institute of Behavioral Neurosciences & Psychology, Fairfield, USA; 3 General Practice, California Institute of Behavioral Neurosciences & Psychology, Fairfield, USA; 4 Pediatrics, California Institute of Behavioral Neurosciences & Psychology, Fairfield, USA; 5 Research, California Institute of Behavioral Neurosciences & Psychology, Fairfield, USA

**Keywords:** non-valvular atrial fibrillation, end-stage renal disease, oral anticoagulation, ischemic and hemorrhagic stroke, stroke under 65, left atrial appendage occlusion, anticoagulant efficacy, stroke prevention, chronic kidney disease, atrial fibrillation

## Abstract

Over the last few years, there has been a rising incidence of atrial fibrillation and chronic kidney disease cases. Stroke is the major complication seen in such patients. The combination of both diseases makes patient management more tedious.

PubMed and Google Scholar underwent screening with keywords and a Medical Subject Heading (MeSH) combination. The words were “atrial fibrillation,” “chronic kidney disease/chronic renal insufficiency,” “anticoagulation,” “efficacy,” and “left atrial appendage occlusion.”

Articles had screening and appraisal. With the English language as a filter, papers from 2002 to 2022 are part of this review. We reviewed studies including male patients with atrial fibrillation and chronic kidney disease under 65 years to see their risk-benefit from anticoagulation. In addition, left atrial appendage occlusion (LAAO) is also compared. A total of eight articles are part of this systematic review.

Age plays a more prominent role than gender regarding the impact of drugs on stroke prevention. LAAO also shows a better outcome than oral anticoagulation, provided people agree to undergo surgery. More studies must be done for this target population, especially comparing results with LAAO and oral anticoagulation.

## Introduction and background

**Figure 1:**
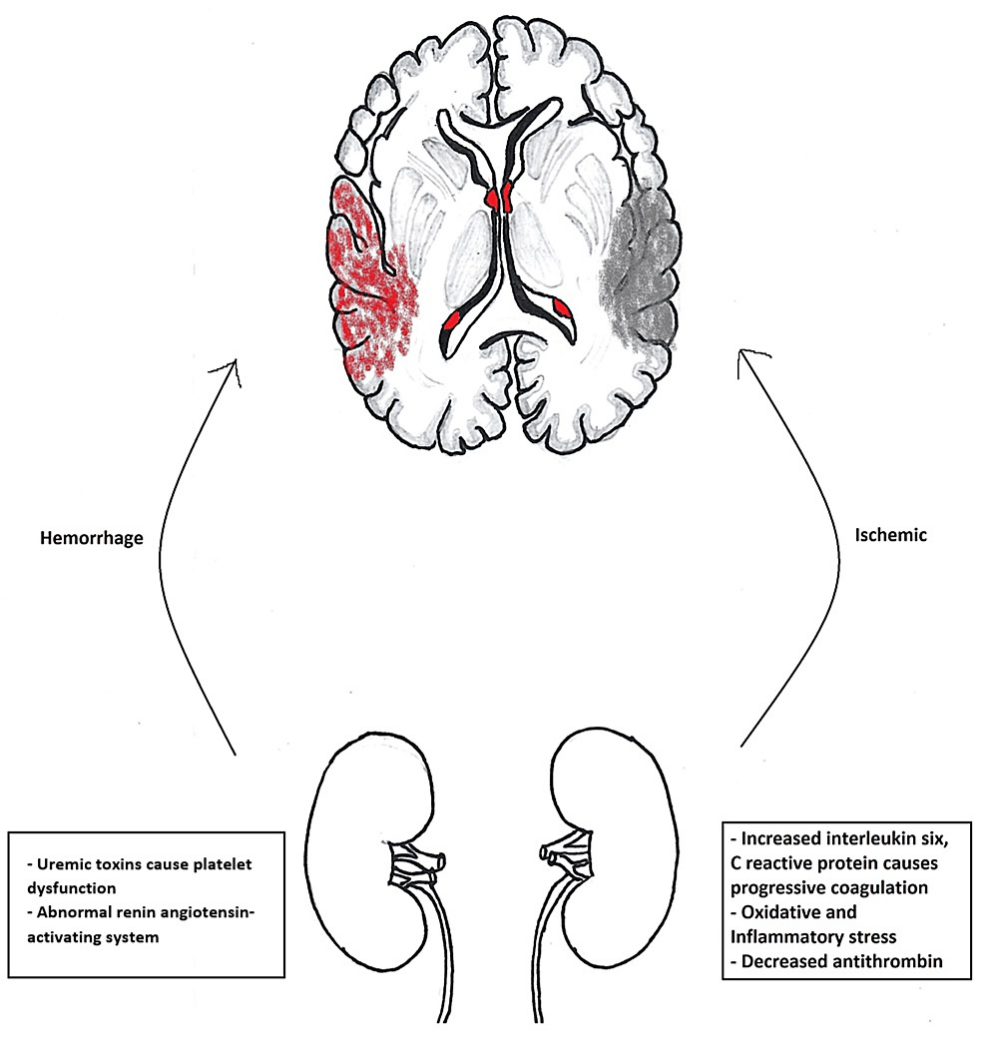
Mechanism of stroke in chronic kidney disease Image credit: Dr. Niriksha Ravi, the corresponding author of the current study.

Stroke is a condition that occurs when there is a diminished blood supply to the brain. Strokes can either be ischemic (85%) or hemorrhagic (15%). Ischemic strokes occur with thrombi formation or when an embolus has dislodgements, causing a vascular block and eventually leading to a stroke caused by hypoperfusion. Hemorrhagic stroke is a local bleed that leads to diminished blood flow to the brain [1]. Patients with atrial fibrillation have five times more risk of having a stroke, and chronic kidney disease patients have a 3.7 times more chance of the same. Additionally, end-stage renal disease cases have 5.8 times more risk than the regular population [2]. Atrial fibrillation (Afib) is defined as abnormal electrical activity in the heart causing an altered cardiac rhythm, distorting normal contraction, and creating a risk for thrombi and subsequent ischemic strokes. Chronic kidney disease (CKD) is associated with protein loss and inflammatory changes as well as a defective coagulation mechanism that increases the risk of bleeding and causes hemorrhagic strokes and thromboembolism, leading to ischemic strokes [3]. The mechanism is depicted in Figure 1.

CKD patients undergoing dialysis have an added bleeding tendency, creating an added threat of hemorrhagic stroke [4]. Patients with an advanced case of CKD and Afib are at an increased risk of bleeds and strokes, making anticoagulation treatment critical for managing such patients [2-4]. The CHA_2_DS_2_-VASc (congestive heart failure [one point], hypertension [one point], age > 75 [two points], diabetes [one point], stroke/transient ischemic attack/thromboembolism [two points], vascular disease [one point], age 65-74 [one point], and sex category - female [one point]) helps determine the need for anticoagulation among Afib patients, and a score of more than one needs immediate oral anticoagulation [5,6]. Long-term anticoagulation is the known way for treating patients with stroke; however, no concrete modality for managing patients with CKD posing a stroke threat is mentioned anywhere [5,7]. Almost 50% of CKD with associated Afib have a massive risk of the thrombi getting dislodged and forming embolic foci in the brain leading to subsequent strokes [6].

For patients with only Afib, warfarin was initially chosen as a treatment drug, but oral anticoagulants were later used to reduce the need for monitoring. Drugs like dabigatran, rivaroxaban, apixaban, and edoxaban are now first-line drugs for the ailment [7]. The drugs used depend on the severity of CKD. In mild cases, warfarin or oral anticoagulants were used, with the latter being the better choice. In severe cases, apixaban is the drug of choice for its safety profile and efficacy. A combination of both conditions poses a greater risk for thrombi formation and an added bleeding risk, making anticoagulation decisions based on risk assessment [5]. Some studies suggest left atrial appendage closure as a modality of treatment in the future [3,5]. Others state that usage of warfarin, rivaroxaban, dabigatran, and other oral anticoagulants may be the best modality for managing patients with combined conditions [8-10]. Until now, no conclusive assumption has been made on a drug of choice, especially in cases with a lower risk for stroke. This systematic review attempts to deduce if anticoagulation can effectively manage the cases of Afib and CKD in patients aged lower than the cut-off range of CHA_2_DS_2_-VASc (19-64 years) and of male gender. The criteria try to assess whether anticoagulation for stroke prevention can provide any benefit in cases with reduced risk factors. This study also attempts to deduce the best possible option for the treatment of such low-risk cases by comparing several anticoagulants with left atrial appendage occlusion (LAAO).

## Review

Methods

Guidelines

Preferred Reporting Items for Systematic Review and Meta-Analysis (PRISMA) 2020 guidelines were analyzed and used to formulate findings and conduct this systematic review [11].

Search Databases

Databases such as PubMed, PubMed Central, MEDLINE, and Google Scholar were used.

Search Strategy

The above databases were initially screened from June 6 to June 13, 2022, with individual keywords followed by key term combinations including “atrial fibrillation,” “chronic kidney disease/chronic renal insufficiency,” “anticoagulation,” “efficacy,” and “left atrial appendage.”

The following Medical Subject Headings (MeSH) search strategy combines key terms with Boolean AND to maximize search accuracy. (“Atrial Fibrillation/analysis"(Majr) OR "Atrial Fibrillation/complications"(Majr) OR "Atrial Fibrillation/drug therapy"(Majr) OR "Atrial Fibrillation/epidemiology"(Majr) OR "Atrial Fibrillation/etiology"(Majr) OR "Atrial Fibrillation/mortality"(Majr) OR "Atrial Fibrillation/prevention and control"(Majr) OR "Atrial Fibrillation/rehabilitation"(Majr) OR "Atrial Fibrillation/therapy"(Majr)) AND (“Chronic kidney disease/chronic renal disease/chronic renal insufficiency” OR "Renal Insufficiency, Chronic/analysis"(Majr) OR "Renal Insufficiency, Chronic/diagnosis"(Majr) OR "Renal Insufficiency, Chronic/drug therapy"(Majr) OR "Renal Insufficiency, Chronic/epidemiology"(Majr) OR "Renal Insufficiency, Chronic/etiology"(Majr) OR "Renal Insufficiency, Chronic/mortality"(Majr) OR "Renal Insufficiency, Chronic/prevention and control"(Majr) OR "Renal Insufficiency, Chronic/rehabilitation"(Majr) OR "Renal Insufficiency, Chronic/therapy"(Majr)) AND ("Anticoagulants/adverse effects"(Majr) OR "Anticoagulants/drug effects"(Majr) OR "Anticoagulants/therapeutic use"(Majr) OR "Anticoagulants/therapy"(Majr) OR "Anticoagulants/toxicity"(Majr)).

Inclusion/Exclusion Criteria

Studies in the English language from 2002 to 2022 were included, and we reviewed articles that included only male patients under 65 years.

Results

**Figure 2:**
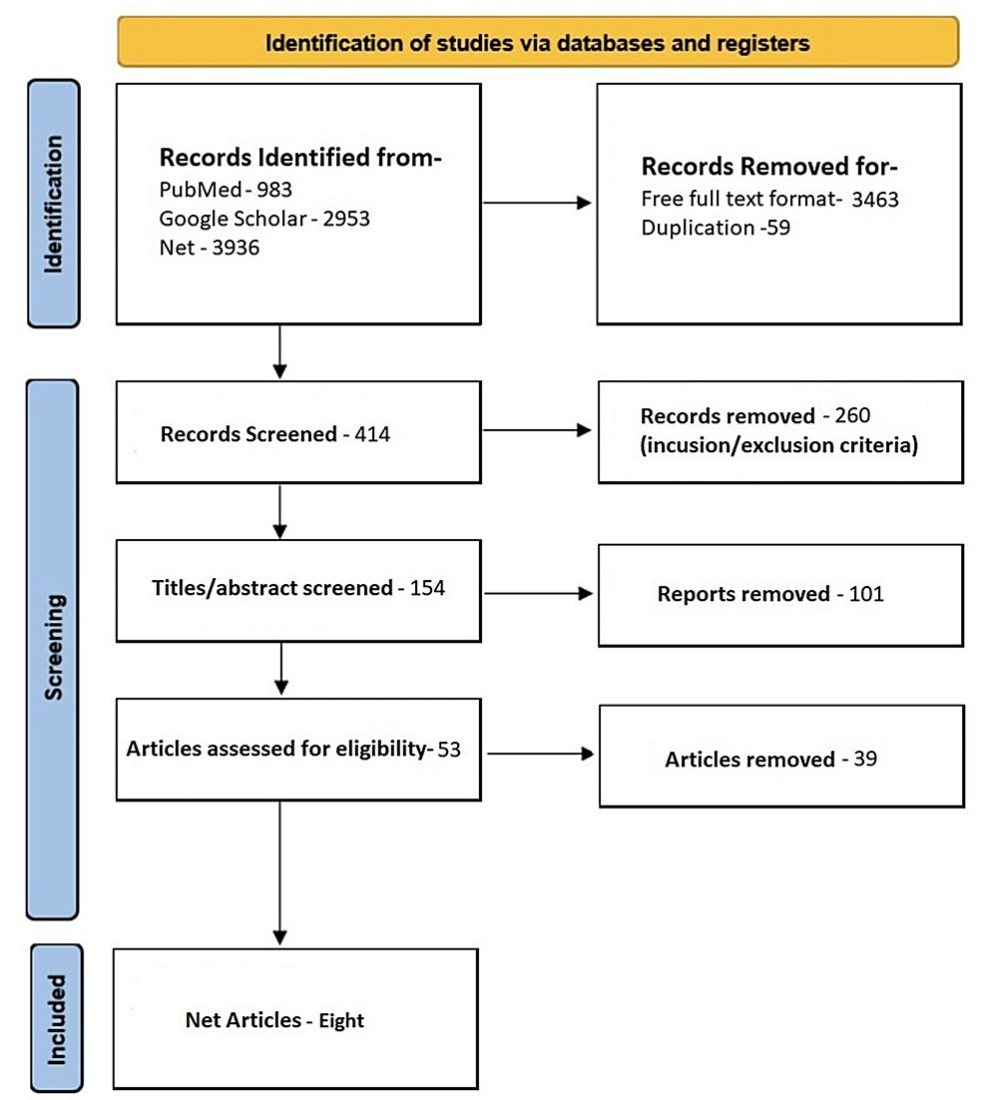
Flowchart of the PRISMA PRISMA: Preferred Reporting Items for Systematic Reviews and Meta-Analyses.

The total number of articles across all databases is 3936. All duplicated articles are removed after the articles are subjected to a selection of only free full texts. Following that, articles are fairly narrowed and screened by their titles or abstracts using the inclusion/exclusion criteria. Finally, articles underwent quality assessments, and the eligible reports came down to a total of eight articles [3,5,7,8-10,12,13]. Figure 2 shows an in-depth overview of the process adopted, namely identification, screening, and the inclusion of articles.

These eight articles included two systematic reviews, one observational study, four literature reviews, and one cohort study as depicted in Table 1. We used Preferred Reporting Items for Systematic Review and Meta-Analysis (PRISMA) 2020 guidelines for systematic reviews, NewCastle-Ottawa Tool for the cohort study and observational study, and Scale for the Quality Assessment of Narrative Review Articles (SANRA) for literature reviews. While reviewing articles, we graded papers as good for this systematic review.

**Table 1:** Overview of articles used and their screening

First author	Year	Type of study	Size
Randhawa et al. [3]	April 2020	A systematic review of 15 studies	47,480
Bhatia et al. [5]	September 2018	Literature review	-
Kaatz and Mahan [7]	July 2014	Literature review	-
Buckley et al. [8]	October 2016	Literature review	-
Lin et al. [9]	April 2021	A cohort study of Taiwanese population	3,358
Coleman et al. [10]	August 2019	Literature review	72,599
Michlicka-Kłyś et al. [12]	April 2022	Single-center, prospective observational study	272
Zhang et al. [13]	April 2020	A systematic review of seven studies	-

Discussion

As the mechanism of stroke evolution in patients with Afib and CKD is understood, different treatments have emerged over the years to help patients with this combination of diseases. Nonetheless, it is still unclear whether these patients will benefit from the treatment. By analyzing information on the possible treatment options, hazard ratio (HR) or standard hazard ratio (SHR), we checked for any benefit in the targeted low-risk study population. Comparison between oral anticoagulants and LAAO helps deduce the best possible treatment option.

Role of Anticoagulation for the Study Population

Oral anticoagulants are the novel choice for Afib and patients with CKD with proven benefits [5,7]. However, when it comes to patients with both comorbidities, the outcomes are based on a benefit-to-risk calculation [5].

A systematic review studying the effects of warfarin for patients with Afib and CKD depending on its stages shows no statistically significant reduction in ischemic strokes for patients (HR: 0.96 for confidence interval [CI]: 0.82-1.13); however, it shows an increased risk for hemorrhagic strokes (HR: 1.49 at 95% CI: 1.03-1.94). However, patients showed no risk for other major bleeds or added mortality [3].

A recent cohort study compares the effects of warfarin and rivaroxaban for patients with Afib and CKD. It shows that before the inverse probability of treatment weighting (IPTW), which is used to balance baseline characteristics of study groups, the male gender has a standard mean deviation (SMD) of 0.08 and 0.18 after IPTW making it statistically significant, thereby showing that the male gender showed significant effects with rivaroxaban over warfarin. Prior to IPTW, the average age limit was higher, but after IPTW, it dropped to a lower stroke risk category. It became 0.09 SMD from an original 0.49. The study shows that the study population’s age acted as a more significant factor for rivaroxaban versus warfarin efficacy. There was no significant effect for ischemic or hemorrhagic stroke or major bleeding, but rivaroxaban reduced the composite endpoint of stroke/embolism (p = 0.02) and gastrointestinal (GI) bleeds (p = 0.01). At a high dose, rivaroxaban significantly reduces ischemic stroke (p = 0.03). Major bleeding case numbers are similar in both groups, but rivaroxaban has a more beneficial effect on ischemic stroke patients (SHR = 0.36). It also has a lesser rate of GI bleeds comparatively (SHR = 0.56). The elimination of rivaroxaban depends lesser on kidney filtration as compared to both dabigatran and edoxaban, thus raising its scope as a drug for patients with severe renal compromise. Therefore, both drugs have similar outcomes at low doses, but a higher dose of rivaroxaban shows superiority over warfarin, with notably significant results for our study group [9]. Another cohort study comparing similar parameters showed that the male patients had an SMD of 0.09 before IPTW and 0.01 after IPTW, which makes it statistically significant, thereby showing that the male gender shows significant effects with rivaroxaban over warfarin. Before IPTW, age < 65 (two groups of 18-49 years and 50-64 years) had an SMD of 0.16, 0.27, and 0.01, 0.02 after IPTW making it statistically significant, thereby showing that lower age shows more significant effects with rivaroxaban than warfarin. Rivaroxaban has a 19% (95% CI: 13%-25%) lesser risk for acute kidney injury (rates: 4.91 versus 8.45) and 18% lesser chance (95% CI: 9%-26%) for stage five CKD progression or hemodialysis (rates: 2.67 versus 4.12). Rivaroxaban has way lesser renal adverse outcomes when compared to warfarin for patients with non-valvular atrial fibrillation (NVAF) [10].

Another review article studying various direct oral anticoagulants (DOACs) shows superiority in its use for mild-moderate cases of Afib and CKD over those using warfarin. In end-stage cases, some studies support the use of warfarin, while other studies state that it does more harm than good. Both dabigatran and rivaroxaban may induce renal injury and raise the risk of GI bleeds. Still, dabigatran is cleared off much faster and increases drug dosage in patients with compromised renal function. On the other hand, apixaban is the least cleared and causes minimal bleeding, making it the drug of choice for such patients. Edoxaban is another alternative that can be used instead of apixaban [5]. Another review article comparing warfarin and several oral anticoagulants stated that apixaban and edoxaban show no change in efficacy with age, unlike dabigatran and rivaroxaban, which causes bleeds in older patients. Apixaban shows no significant difference in elimination with renal functioning, making it ideal for patients with end-stage renal disease (ESRD) or on hemodialysis. In the study population, apixaban and warfarin show no difference in the efficacy of stroke prevention and bleeding risk. As their kidneys start to fail with age, older patients are more vulnerable to toxicity. Dabigatran is highly dependent on renal functioning; thus, a failing kidney raises its concentration. Comparatively, rivaroxaban poses less risk for toxicity with failing kidneys. Men also showed a much faster drug clearance rate than women, increasing their risk for drug side effects [8].

With deteriorating renal function, especially with age, the doctor must adjust drug dosage to prevent adverse effects like counteractive bleeds. Apixaban needs the slightest adjustment since apixaban excretion is independent of renal function, unlike rivaroxaban or dabigatran, so they need maximum adjustment for dosage. Warfarin use shows reduced strokes and thromboembolism for cases having Afib and CKD (hazard ratio: 0.76); on the other hand, acetylsalicylic acid has increased risk (hazard ratio: 1.17). Despite the need for monitoring and more bleeding risk for CKD patients, doctors use warfarin as it is independent of renal function for excretion, especially in creatinine clearance (Crcl) of 25-30. Most patients studied for various drugs were of the male gender, thereby showing its potential role for the benefit [7]. Table 2 provides a summary of all the articles discussed in this section.

**Table 2:** Summary of articles used

First author	Publication date/type of study	Purpose	Results
Bhatia et al. [5]	September 2018, literature review	To overview the options for atrial fibrillation and chronic kidney disease to reduce thrombo-embolism based on chronic kidney disease stages	Different stages of chronic kidney disease need special drug usage for their management.
Kaatz and Mahan [7]	July 2014, literature review	To study the anticoagulant mechanism for stroke prevention in atrial fibrillation cases, especially with varying kidney functions	Avoid novel oral anticoagulants when the creatinine clearance is less than 25-30 mL/min due to limited data about their usage. One may choose warfarin in severe cases but at lower dosages.
Randhawa et al. [3]	April 2020, systematic review	To analyze warfarin and its role in atrial fibrillation and end-stage renal disease	Warfarin causes no significant change in ischemic stroke risk (hazard ratio: 0.96), has a higher hemorrhagic stroke rate (hazard ratio: 1.49), and has no significant effect on the risk of major bleeding or mortality.
Lin et al. [9]	April 2021, cohort study	To compare rivaroxaban and warfarin for atrial fibrillation and end-stage renal disease cases	Bleeding risk had no statistically significant difference. Rivaroxaban was significant for a reduction in ischemic stroke rate when compared to warfarin.
Coleman et al. [10]	August 2019, literature review	To study the effects of rivaroxaban compared to warfarin on kidney function in non-valvular atrial fibrillation	Rivaroxaban has 19% lesser (95% CI: 13%-25%) acute kidney episodes (rates = 4.91 vs 8.45) and 18% reduced need for dialysis or chronic kidney disease progress.
Buckley et al. [8]	October 2016, literature review	To study the role of novel oral anticoagulants in atrial fibrillation, which depends on the stage of chronic kidney disease, weight, or age of the patients	Apixaban is the best drug in renal compromise, followed by edoxaban. Dabigatran and rivaroxaban are less opted for older patients.

Effect of Various Novel Oral Anticoagulants (NOACs) Compared to Left Atrial Appendage Closure for Study Population

**Figure 3:**
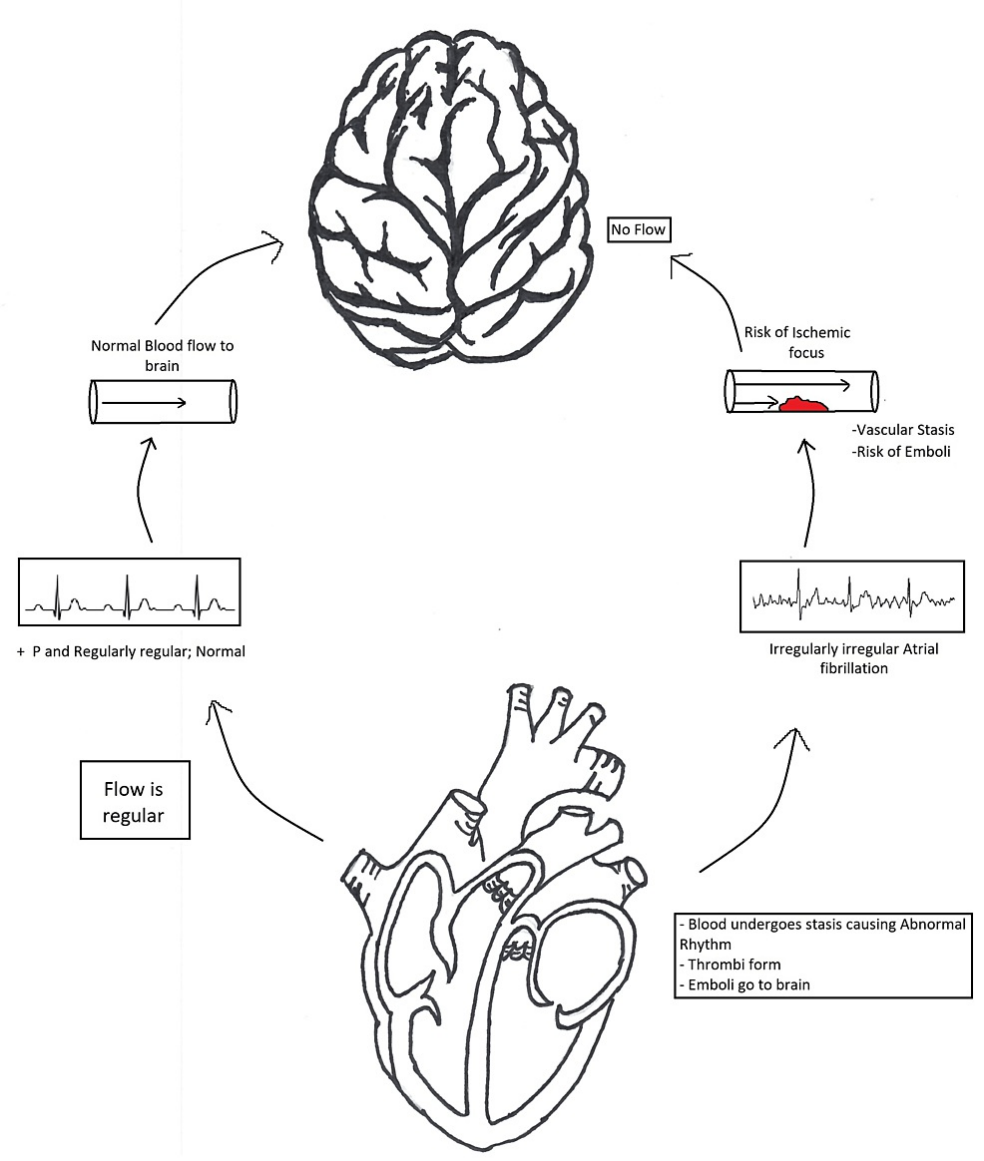
Stroke evolution in atrial fibrillation Image credit: Dr. Niriksha Ravi, the corresponding author of the current study.

A newly emerging approach over these years has been the LAAO for patients with Afib and CKD in NVAF cases [12]. Figure 3 explains the mechanism of stroke in atrial fibrillation.

The left atrial appendage (LAA) contributes to about 91% of thrombi in cases of NVAF. The majority of targeted patients are of the higher age group, putting them at risk according to stroke assessment criteria. However, men are more affected by the patient load, which is low-risk gender for strokes. About 98% of patients with combined CKD and Afib benefited from the procedure; 1.9% had ischemic strokes, and 1% had hemorrhagic strokes. Also, 96.4% benefited from the lower chance of ischemic stroke, and 97.9% benefited from the reduction in hemorrhagic stroke. A recent cohort shows a statistically significant effect for reduced thrombi evolution risk (p = 0.03) and no significant bleeding complications (p = 0.768). Unlike NOAC, LAAO has more evidence for severe CKD [12]. Regarding rivaroxaban, 6.2% had ischemic and hemorrhagic strokes, and after using warfarin, 9.8% of patients had ischemic and hemorrhagic strokes [9].

LAAO devices could be a promising alternative for patients with severe CKD (like the last stage) and Afib for stroke prevention if they cannot tolerate anticoagulation. It shows a better outcome than warfarin, which has no significant effect in cases of ischemic strokes. In addition, warfarin also raises the risk of hemorrhagic strokes (HR: 1.49) [3].

After examining the use of LAAO in Afib and CKD, it is reasonable to conclude that it is safe for Afib and CKD patients, especially for those needing long-term management. In patients on anticoagulants, one can easily stop using them before surgery. Adverse events in the perioperative period after LAAO is generally low, with events like pericardial effusion/tamponade (IR: 1.90%) and mortality rate (1.10%). During the follow-up period, the incidences of stroke/transient ischemic attack and bleeding are 2.17% and 4.53%, respectively. Patients fitted with LAAO devices do not require prolonged oral anticoagulants; many only need single antiplatelet therapy or no therapy, thus increasing compliance. Men are the main targeted population. Renal function is not affected. Contrast-induced acute kidney injury (CI-AKI) is an important complication resulting from intravascular administration of contrast media and is likely to occur in CKD patients. The all-cause mortality rate for oral anticoagulants to prevent embolic events in Afib with CKD stage three is 5%-6%. Though male populations are at risk in most LAAOs, the majority of patients are over the age of 70. Thus, it could be a safer option for younger groups. More studies need to be done on this matter to opine further [13]. Table 3 provides a summary of all the articles discussed in this section.

**Table 3:** Summary of the articles

First author	Date of publication/type of study	Purpose	Results
Michlicka-Kłyś et al. [12]	April 2022, prospective observational study	To see the efficacy of left atrial appendage occlusion for cases with chronic kidney disease and non-valvular atrial fibrillation over the course.	Left atrial appendage occlusion has a relative risk reduction of 96.4% for strokes and a relative risk reduction of 97.9% for bleeding.
Lin et al. [9]	April 2021, cohort study	To compare rivaroxaban and warfarin for atrial fibrillation and end-stage renal disease cases.	Bleeding risk had no statistically significant difference. Rivaroxaban was significant for a reduction in ischemic stroke rate when compared to warfarin.
Randhawa et al. [3]	April 2020, systematic review	To analyze warfarin and its role in atrial fibrillation and end-stage renal disease.	Warfarin causes no significant change in ischemic stroke risk (hazard ratio: 0.96), has a higher hemorrhagic stroke rate (hazard ratio: 1.49), and has no significant effect on the risk of major bleeding or mortality.
Zhang et al. [13]	April 2020, systematic review	To study the left atrial appendage occlusion safety profile and performance in left atrial appendage occlusion and chronic kidney disease.	Low complications in the perioperative period and no kidney impact. It has high safety and low rates of strokes to transient ischemic attack and bleeds.

Limitations

There are several limitations to our study. We only selected the studies in English that qualified as a free full-text format. We only included articles published in the last 20 years, limiting access to older papers. In addition, only two major databases helped deduce the results. Apart from this, a major limitation is the scarcity of papers available to compare the study's parameters.

## Conclusions

With an increase in the emerging cases of strokes in patients having atrial fibrillation and CKD, an attempt is made to analyze a pool of patients from various studies that are believed to be having a lower stroke risk. The lower age group plays a more significant role in the beneficial effect of drugs than the male gender. In addition, there is a comparison between the rates of adverse effects of LAAO and oral anticoagulation. For patients agreeing to undergo an intrusive procedure, LAAO has better outcomes than oral anticoagulants like apixaban, which is the most opted drug for this group. It has also been shown to be better for long-term management; however, being a surgical procedure, it is a less opted method. LAAO can only be used in some instances, thereby restricting its usability. However, it can offer a possible ideal future option for those with ESRD and atrial fibrillation of the non-valvular type. More studies need to be done for this low-risk group to further assess anticoagulation’s benefits in patients with these conditions. LAAO and oral anticoagulation comparison studies also need to be done to a further extent in this population.
